# Visualized Analysis of Adolescent Non-Suicidal Self-Injury and Comorbidity Networks

**DOI:** 10.3390/bs15111513

**Published:** 2025-11-07

**Authors:** Zhen Zhang, Juan Guo, Yali Zhao, Xiangyan Li, Chunhui Qi

**Affiliations:** 1 Faculty of Education, Henan Normal University, Xinxiang 453007, China; zhangzhen201912@htu.edu.cn (Z.Z.); 2310084002@stu.htu.edu.cn (J.G.); 2310283142@stu.htu.edu.cn (Y.Z.); 2410084005@stu.htu.edu.cn (X.L.); 2 Faculty of Education, Henan University, Kaifeng 475001, China

**Keywords:** non-suicidal self-injury, adolescents, comorbidity, visualization analysis

## Abstract

Non-suicidal self-injury (NSSI) has become an increasingly salient mental health concern among adolescents, and it commonly co-occurs with depression, anxiety, borderline personality disorder, substance use, and childhood maltreatment, forming a complex psychological risk structure. Despite a growing body of literature, a systematic understanding of the structural links between NSSI and psychiatric comorbidities remains limited. This study uses bibliometric and visualization methods to map the developmental trajectory and knowledge structure of the field and to identify research hotspots and frontiers. Drawing on the Web of Science Core Collection, we screened 1562 papers published between 2005 and 2024 on adolescent NSSI and comorbid psychological problems. Using CiteSpace 6.3.R1, VOSviewer 1.6.20, and R 4.3.3, we constructed knowledge graphs from keyword co-occurrence, clustering, burst-term detection, and co-citation analyses. The results show an explosive growth of research in recent years. Hotspots center on comorbidity mechanisms of mood disorders, the impact of childhood trauma, and advances in dynamic assessment. Research has evolved from describing behavioral features toward integrative mechanisms, with five current emphases: risk factor modeling, diagnostic standard optimization, cultural sensitivity, stratified intervention strategies, and psychological risks in special populations. With big data and AI applications, the field is moving toward dynamic prediction and precision intervention. Future work should strengthen cross-cultural comparisons, refine comorbidity network theory, and develop biomarker-informed differentiated interventions to advance both theory and clinical practice.

## 1. Introduction

Non-suicidal self-injury (NSSI) refers to deliberate damage to one’s own body tissue—such as cutting, burning, and scratching—in the absence of intent to die, with a typical onset in early adolescence (Zetterqvist, 2015). Unlike suicidal behavior, NSSI is primarily motivated by relief from negative affect or by the pursuit of emotional release. In recent years, its prevalence has risen globally and now constitutes a serious public health concern (De Luca et al., 2023). International estimates suggest a prevalence of around 17–18% among adolescents (Kaess et al., 2019), and a meta-analysis of non-clinical youth samples reported comparable lifetime and 12-month rates of 22.0% (Xiao et al., 2022). NSSI commonly co-occurs with other psychological problems, including depressive and anxiety disorders, borderline personality disorder, and substance use (Buelens et al., 2020; Menderes & Çuhadaroğlu, 2025; Niu et al., 2024). Specific forms of childhood maltreatment—especially emotional and sexual abuse—substantially increase risk; early identification and intervention for children exposed to such maltreatment can minimize the likelihood of NSSI in adolescence (Xie et al., 2024). NSSI also elevates suicide risk. Compared with peers without NSSI, adolescents who engage in NSSI face up to a 30-fold higher risk of suicide attempts (Usmani et al., 2024). These findings underscore the gravity of NSSI and the need to probe its key risk factors and psychological mechanisms so as to inform evidence-based prevention and intervention. However, most studies focus on links between NSSI and single disorders in specific samples or cultural settings, and a systemic perspective on the overall structure of adolescent comorbidities remains lacking.

With the rapid growth of publications, bibliometrics and visualization now provide new tools for understanding adolescent NSSI comorbidity networks by mining keyword co-occurrence patterns, thematic clusters, and evolutionary trends to construct knowledge maps of the field. They can not only deeply analyze the existing literature but also accurately locate the fields that need to be further explored and point out the direction for subsequent research (Öztürk et al., 2024). Using the Web of Science Core Collection, we applied bibliometric and visualization techniques to papers from 2005 to 2024 on adolescent NSSI and psychiatric comorbidity. Analyses of 1562 records aim to (1) identify structural relations between NSSI and major comorbid mental health problems; (2) reveal dominant and emerging hotspots; and (3) offer theoretical references and future directions for multidisciplinary research and clinical interventions.

## 2. Materials and Methods

### 2.1. Data Sources

**Figure 1:**
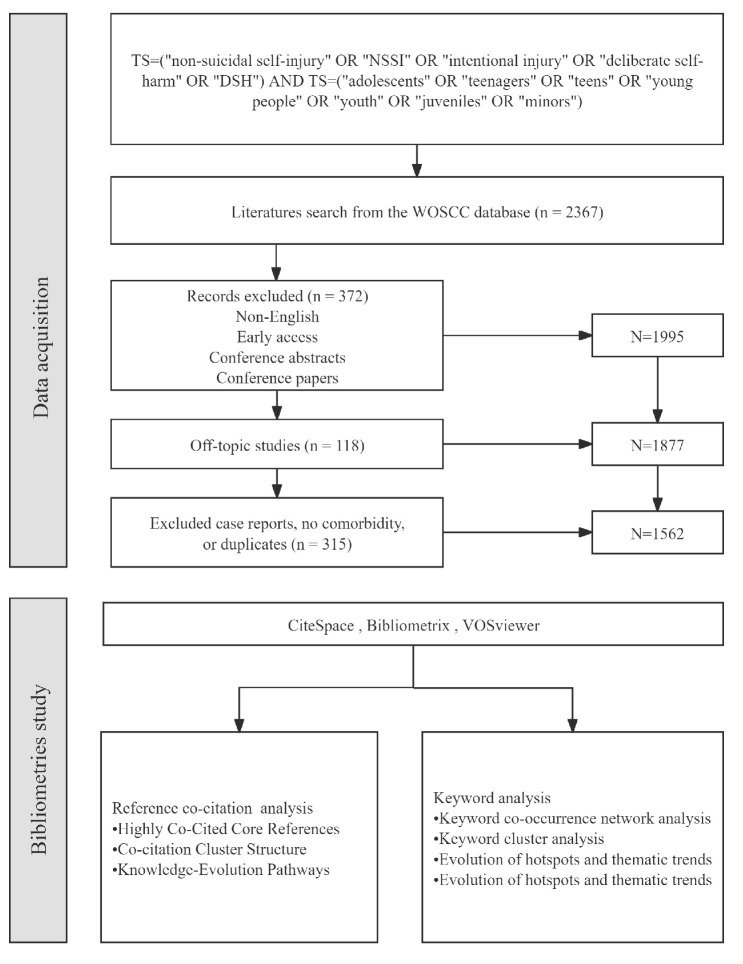
Structure of the article.

English-language papers were retrieved from the Web of Science Core Collection between 1 January 2005 and 31 December 2024 to capture trends in adolescent NSSI and comorbid psychological problems. An advanced topic query was used: TS = (“non–suicidal self–injury” OR “NSSI” OR “intentional injury” OR “deliberate self–harm” OR “DSH”) AND TS = (“adolescents” OR “teenagers” OR “teens” OR “young people” OR “youth” OR “juveniles” OR “minors”). Following database retrieval, two reviewers independently screened titles and abstracts for potential eligibility, after which the full texts of candidate records were assessed against the inclusion criteria. Any discrepancies were resolved through discussion until a consensus was reached. Language was limited to English and document types to articles and reviews, yielding 1995 records. Manual screening then applied the following inclusion/exclusion criteria: participants aged 10–19; NSSI clearly defined as lacking suicidal intent; and at least one comorbid problem (e.g., depression, anxiety, trauma, substance use, personality disorder), and case reports were excluded. The final analytic set comprised 1562 English-language papers. The structure of the article is shown in Figure 1.

### 2.2. Research Methods and Tools

Records exported as plain text from the WoSCC were analyzed using three bibliometric tools—CiteSpace 6.3.R1, Bibliometrix 4.1.0 (with Biblioshiny), and VOSviewer 1.6.20—to visualize annual publications, keywords, and co-cited references. CiteSpace 6.3.R1 excels at revealing research frontiers via burst-term analyses, co-citation clusters, timelines, and timezone views; Bibliometrix/Biblioshiny supports robust bibliometric statistics, thematic evolution, and strategic maps; and VOSviewer 1.6.20 builds co-occurrence and collaboration networks of authors, institutions, and keywords with clear, aesthetic graphics.

## 3. Results

### 3.1. Annual Publication Trends

**Figure 2:**
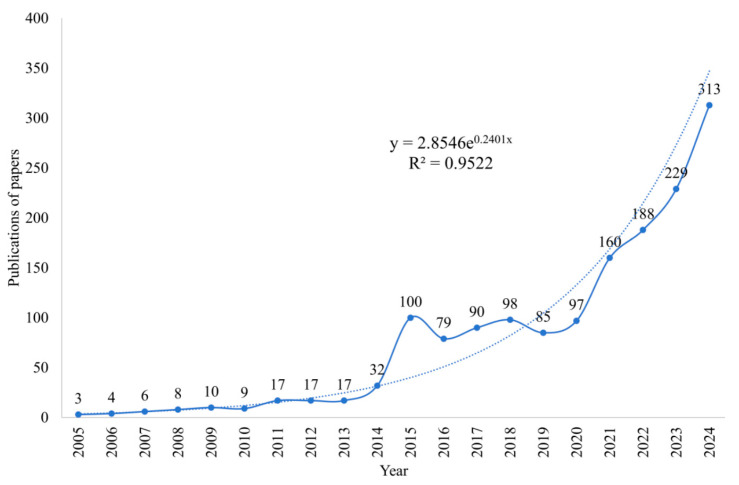
Trends in the number of publications on NSSI from 2005 to 2024.

Publication counts are key indicators of a field’s developmental level and scholarly impact, reflecting research intensity, sustainability, and the degree of academic attention (Ale Ebrahim et al., 2014). As shown in Figure 2, the number of publications in this domain exhibits a clear upward trajectory. From 2005 to 2013, annual output remained relatively low—generally ranging from single digits to just over a dozen papers—with slow growth, indicating that the field was still in its initial exploratory stage. Beginning in 2014, research activity rose markedly; in 2015, the annual count reached 100 for the first time, representing a leap from the previous year and signaling entry into an accelerated growth phase. It can be mainly attributed to a key progress in the standardization of diagnosis in this field, that is, DSM–5 lists non-suicidal self-injury as a disease unit to be further studied, which provides a key unified framework for empirical research (Zetterqvist, 2015). During 2016–2020, although year-to-year counts fluctuated slightly, the overall level remained high, demonstrating steady and sustained development. Since 2021, the field has experienced explosive growth: 160 publications in 2021, 188 in 2022, 229 in 2023, and 313 in 2024—18.4 times the 2013 figure—highlighting the prominence and continued momentum of adolescent NSSI research in mental health and public health. Moreover, the fitted curve yields a goodness of fit of R^2^ = 0.9522, indicating a highly consistent exponential growth pattern and suggesting substantial potential for further development and expansion. The exponential growth after 2021 is driven by the integration of external and internal factors. The global novel coronavirus epidemic has constituted a major public health crisis, which has sharply increased the mental health needs of adolescents and the urgency of research. At the same time, the maturity and application of new methods, such as big data analysis and network analysis, have opened up a new research path for this field.

### 3.2. Hot Topics

#### 3.2.1. Keyword Co-Occurrence Network Analysis

**Figure 3:**
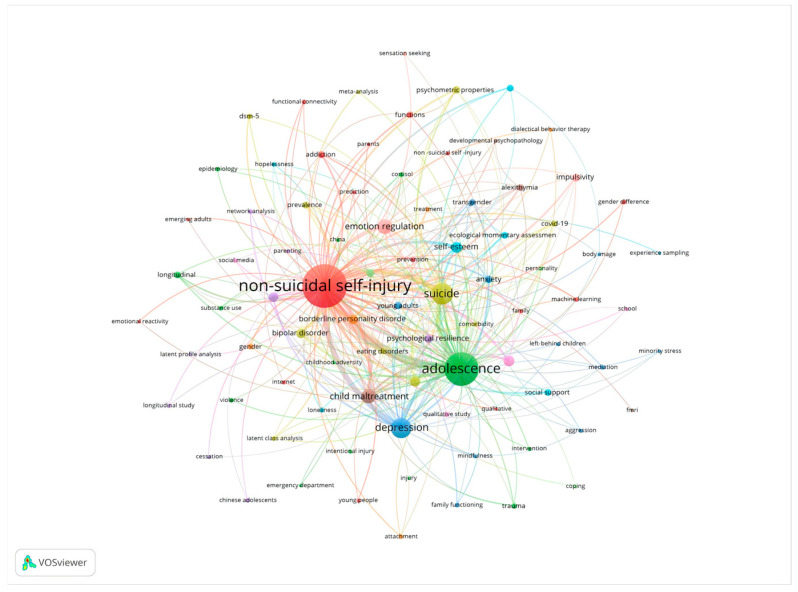
Keyword co-occurrence map.

To identify the major psychiatric comorbidities involved in adolescent non-suicidal self-injury (NSSI) research and their structural relations, we employed VOSviewer 1.6.20 to conduct a keyword co-occurrence analysis of the relevant literature and to construct a visual network map. In the co-occurrence network, larger nodes indicate higher term frequency, reflecting greater attention within the field. Centrality indexes the importance of a keyword node in the network; terms with high betweenness centrality act as bridges in the literature and occupy pivotal positions in the domain. Keyword co-occurrence analysis not only reveals the logical relationships among research themes but also reflects the field’s research foci and developmental trajectory. Table 1 organizes the high-frequency keywords: “non–suicidal self–injury,” “adolescence,” “suicide,” “prevalence,” and “harm” appear most often and constitute the core thematic set of the field. In the map, these keywords not only occur frequently but are also tightly interlinked, forming a relatively stable knowledge network. In addition, the betweenness centrality values of “mental health,” “borderline personality disorder,” “behavior,” “child maltreatment,” and “symptoms” all exceed 0.05, indicating that they function as bridges connecting different subfields and are key terms driving cross-topic integration. Figure 3 shows that the keyword co-occurrence network has high overall density and close inter-node connections, further indicating a high degree of thematic concentration, a clear structure, and a well-organized knowledge system. Based on the content of the high-frequency keywords, four major hotspot directions can be summarized. First, emotion disorder research centered on “depression,” “anxiety,” and “suicide” underscores the strong association between NSSI and emotion regulation difficulties, highlighting the critical role of negative affect in self-injury. Second, psychopathological comorbidity research—covering “borderline personality disorder,” “eating disorders,” “substance use,” and “impulsivity”—focuses on cross-cutting mechanisms linking NSSI with personality disorders, eating disorders, and substance use disorders. Third, population and experience clusters represented by “adolescence,” “child maltreatment,” and “community sample” attend to age-specific features of youth and the impact of adverse early experiences on NSSI. Fourth, measurement tools and methods organized around “symptoms,” “psychometric properties,” “validity,” and “reliability” emphasize the pivotal role of assessment instruments in NSSI research. Together, these hotspots delineate the current contours of the NSSI knowledge map and provide a solid foundation for subsequent analyses of trend evolution, cluster path identification, and theoretical development.

#### 3.2.2. Keyword Cluster Analysis

**Figure 4:**
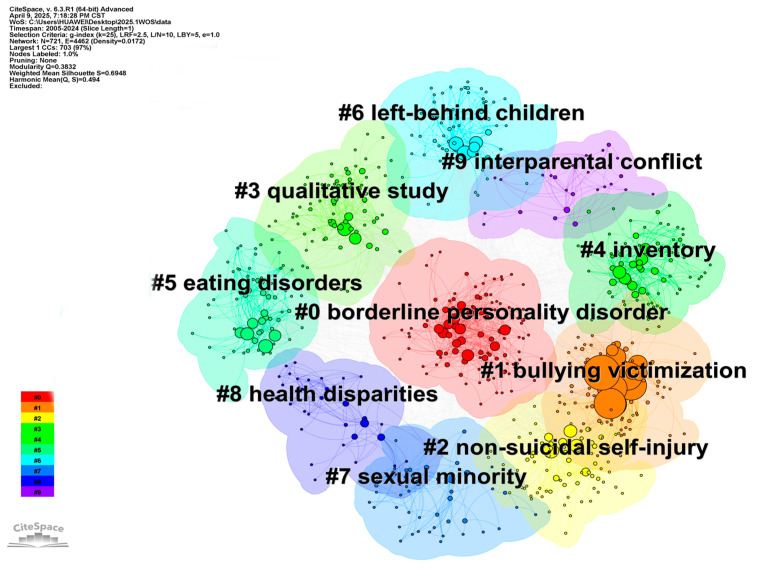
Keyword cluster map.

To further explore hotspot topics and future research directions, we used CiteSpace 6.3.R1 to conduct a cluster analysis of keyword co-occurrence relations in the adolescent non-suicidal self-injury (NSSI) field. As shown in Figure 4, the resulting network comprises 721 nodes and 4462 links. According to the network structure and the clarity of clustering, CiteSpace6.3.R1 provides two indicators: module value (Q = Modularity) and average contour value (S = Weighted Mean Silhouette). When Q > 0.3 and S > 0.5, clustering is considered to be significant and reasonable. In total, 10 clusters were generated; the clustering modularity (Modularity Q) is 0.3832, and the Mean Silhouette (Mean Silhouette S) is 0.6948, indicating a structure with moderately high clarity and good thematic separability and demonstrating strong clustering validity and interpretability. The largest connected component contains 703 nodes, accounting for 97% of all nodes, which further corroborates the network’s overall cohesion and the effectiveness of the analysis. Based on thematic commonalities among the high-frequency keywords and their focal content, the ten clusters can be grouped into four major thematic directions.

(1) Psychopathological mechanisms centered on borderline personality disorder, emotion regulation, and neural mechanisms (#0, #5). This line of work—featuring keywords such as “borderline personality disorder,” “functional connectivity,” “cortisol,” and “eating disorders”—reveals neuropsychological links between NSSI and comorbid conditions (e.g., eating disorders, mood disorders). It provides important clues for understanding the deeper psychological bases of NSSI and lays a theoretical foundation for future integration of cross-dimensional intervention models. As a clinical disorder marked by pronounced affective instability and impulsivity, borderline personality disorder has been shown in multiple ways to significantly increase adolescents’ risk of engaging in NSSI (Reichl & Kaess, 2021); NSSI and BPD symptoms are closely related, with negative emotions such as loneliness, impulsivity, and separation anxiety functioning as bridges between NSSI and BPD. These findings supply new theoretical grounds and intervention leads for early prevention of comorbidity between NSSI and BPD (Buelens et al., 2020). At the neural level, studies using functional magnetic resonance imaging (fMRI) have examined activation patterns in brain regions related to visual processing and emotion regulation among adolescents with NSSI, documenting marked abnormalities in specific areas (e.g., the prefrontal cortex, amygdala, and cingulate gyrus) during emotion processing tasks. Probing alterations in regional brain activity linked to visual and emotional regulation functions can improve understanding of the potential neural mechanisms of adolescent NSSI (Liao et al., 2024). Biomarker research—including cortisol levels—also indicates possible dysregulation in the stress response system among individuals who self-injure; adolescents who engage in NSSI may exhibit a stronger cortisol awakening response, further supporting an integrated biopsychosocial framework for understanding NSSI (Reichl et al., 2016).

(2) Social stress pathways focused on school and family risks (#1, #6, #9). Keywords such as “bullying victimization,” “left–behind children,” and “interparental conflict” point to the predictive role of structural trauma encountered during socialization in the emergence of self-injury. Related studies indicate that experiences of bullying may be an important risk factor for NSSI, with such experiences significantly increasing adolescents’ risks of NSSI and suicidal tendencies in both the short and long term (Serafini et al., 2023). The quality of intimate relationships is closely associated with emotion–regulation capacity and mental health outcomes. A lack of social connectedness to family and school often increases the likelihood that adolescents will adopt NSSI as a coping strategy when facing stressors (e.g., depressive symptoms or bullying); boys and girls may implement NSSI in functionally different ways to relieve distress when relationships with parents are estranged or when school belonging is lacking (Baker et al., 2023). Risk factors for NSSI include psychiatric disorders, bullying, and adverse childhood experiences (ACEs). ACEs—especially emotional and/or sexual abuse—are closely associated with the occurrence of NSSI during adolescence (Calvo et al., 2024). Negative family life events—such as highly expressed emotion among parents, harsh parenting, and poor parent–child communication—also significantly predict NSSI (Wedig & Nock, 2007); distancing from parents due to parental criticism is regarded as a key component in the development of NSSI. Adolescents who engage in NSSI often exhibit lower trust in parents, reduced communication frequency, and greater emotional estrangement; they are more sensitive to parental psychological and behavioral control and report significantly lower satisfaction with parents and lower overall well-being than those without NSSI. Interparental conflict further indirectly predicts NSSI via a chain of mediating effects involving harsh parenting and identity diffusion (Zhong et al., 2024). By contrast, adolescents who are adept at building positive interpersonal relationships are more likely to experience positive affect and a richer, more engaging inner world—factors that serve as protective influences against NSSI.

(3) Social marginalized group psychology focused on identity and group differences (#7, #8). Research on socially marginalized group psychology—centered on identity and group differences—has drawn wide attention in the NSSI field in recent years. Keywords such as “sexual minority,” “transgender,” and “health disparities” highlight the elevated NSSI risk faced by gender/sexual minorities and other disadvantaged groups amid social prejudice and psychological stress. Existing studies show that sexual minority adolescents, particularly bisexual youth, exhibit higher rates of NSSI than both homosexual and heterosexual adolescents (D. Chen et al., 2022; Peters et al., 2020). These findings underscore the need to probe the potential mechanisms linking sexual orientation and NSSI, including the mediating roles of social stigma, internalized oppression, psychological isolation, and identity conflict processes. Building on this, further clarifying the associative pathways among sexual orientation, NSSI, and suicidality will help develop more inclusive and sensitive intervention strategies. Sexual minority youth—including lesbian, gay, bisexual, pansexual, and other non-heterosexual identities—are regarded as a highly vulnerable group in NSSI research. Encouraging the inclusion of these adolescents in targeted mental health prevention programs and policy design is a key public health priority. In addition, gender-based group differences exhibit marked geographic cultural heterogeneity. In North America and Europe, the prevalence of NSSI among female adolescents is roughly twice that among males; however, in Asia, this pattern is reversed, with some studies reporting higher NSSI prevalence among male adolescents than females (Moloney et al., 2024). This suggests that current NSSI epidemiology often reports results by “gender category,” yet still lacks a systematic approach to quantify and explain cross-regional, cross-cultural differences in gendered prevalence. Over the past three decades, significant variation in adolescent NSSI prevalence has persisted across gender, geographic region, and sociodemographic indices worldwide. Research on gender and regional differences from a global perspective is thus crucial for devising gender-sensitive, culturally adaptive intervention strategies (Tan et al., 2025). The elevated risk of NSSI among sexual and gender minority (SGM) populations has been repeatedly documented; however, multilevel social correlates remain underappreciated, and future research and interventions should systematically incorporate structural-level factors (Richards et al., 2025). Such strategies can not only enhance the specificity and effectiveness of NSSI prevention but may also, in the longer term, meaningfully reduce suicide risk among sexual minority and gender marginalized youth.

(4) Measurement and assessment oriented toward method and tool development (#2, #3, #4). Keywords such as “non–suicidal self–injury,” “qualitative study,” and “inventory” reflect a diversified trajectory in NSSI research—from functional models to scale development and from quantitative analyses to qualitative interviews. While scale-based assessments often yield more accurate results, heterogeneity in NSSI definitions and measurement tools has also produced inconsistencies in prevalence estimates. For example, some studies assess self-injury using a single item (e.g., a yes/no question), whereas others adopt multidimensional instruments that cover behavior frequency, functions, injury sites, and maintaining motives (Gillies et al., 2018; Swannell et al., 2014). Early cross-sectional and longitudinal work frequently relied on retrospective assessments of NSSI and related variables, limiting precise understanding of the mechanisms of maintenance, cessation, or exacerbation. In recent years, with advances in methodology and technology, multiform approaches—including ecological momentary assessment (EMA) and comparative dynamic assessment—have been applied (Goreis et al., 2025). By collecting real-time information on individual states, these methods facilitate a more fine-grained capture of the dynamic process of NSSI and its underlying mechanisms (Geng et al., 2022). This methodological diversification is advancing NSSI measurement and assessment from coarse appraisal toward more systematic and granular evaluation, providing solid technical support for further elucidation of the psychological mechanisms of self-injury and the development of intervention strategies.

#### 3.2.3. Evolution of Hotspots and Thematic Trends

**Figure 5:**
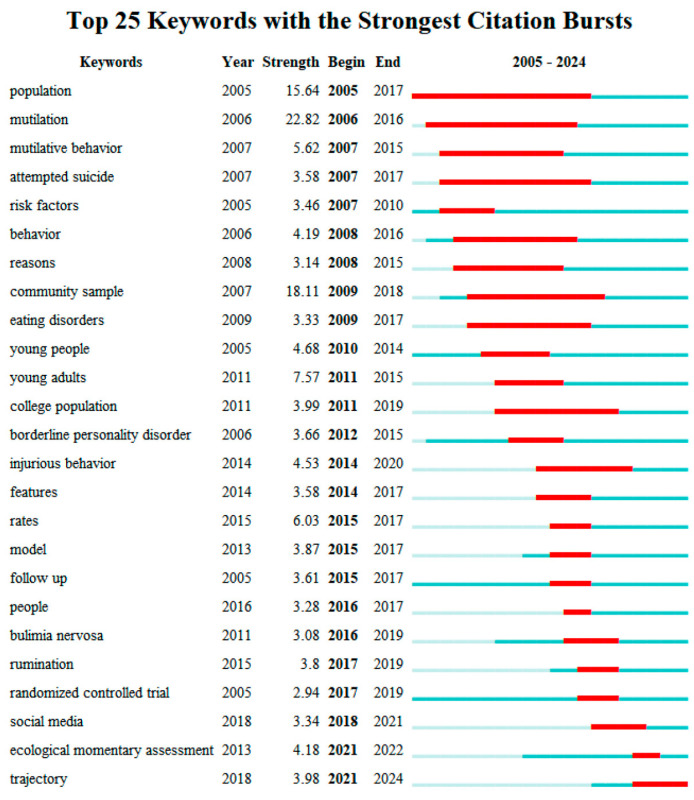
Burst keyword map showing the burst timing and duration of high-frequency keywords in the NSSI field from 2005 to 2024, revealing the evolution of research hotspots across different periods.

To reveal the developmental trajectory and frontier dynamics of adolescent non-suicidal self-injury (NSSI) research, we dynamically tracked high-frequency keywords from 2005 to 2024 using burst-term analysis and a thematic evolution map. Burst-term analysis reflects emerging trends in hotspots and research frontiers across different periods (Geng et al., 2022). By identifying keywords that surge within specific time windows, we can delineate active phases in NSSI scholarship and the pathways of hotspot evolution. The “burst strength” of a term represents the degree of heightened attention it receives during a given interval, whereas the “burst onset and offset” indicate the duration over which the keyword serves as a research focus. Considering both first appearance times and burst windows allows for a multi-dimensional assessment of the field’s renewal speed and research vitality. Figure 5 depicts the 25 most active burst keywords from 2005 to 2024, with overall research exhibiting an “early concentration—mid–stage expansion—late–stage deepening” pattern.

From 2005 to 2010—the stage when NSSI research began and rapidly heated up—keywords such as “population,” “mutilation,” “mutilative behavior,” and “attempted suicide” showed strong bursts, indicating a primary focus on the phenomenon of self-injury, its links to suicide, and epidemiologic features (Nock, 2009; Nock & Favazza, 2009; Nock & Kessler, 2006). Meanwhile, terms like “risk factors,” “behavior,” and “reasons” also displayed burst tendencies, suggesting an initial exploration of the motives and psychological characteristics of NSSI (Glenn & Klonsky, 2009; Klonsky & Glenn, 2008).

After 2010, research topics gradually broadened and diversified. Keywords such as “community sample,” “college population,” and “young adults” began to burst, reflecting a shift from adolescent-only to youth samples and from clinical to community populations (Brickman et al., 2014). In addition, comorbidity-related concepts—“borderline personality disorder” and “eating disorders”—surged, marking growing cross-linkages between NSSI and other psychiatric domains (Turner et al., 2015).

Since 2014, hotspots have clearly tilted toward cognitive mechanisms, emotion regulation, and environmental factors. Bursts in “injurious behavior,” “features,” “rumination,” and “social media” indicate a transition from “behavioral identification” to “mechanistic explanation.” The sustained bursts of “rumination” and “social media” reflect a dual emphasis on adolescents’ internal cognitive processing and the external media ecology (Buelens et al., 2019, 2020; Hasking et al., 2019; Nesi et al., 2021). This trajectory aligns with the expanding need in mental health research to account for influences on adolescent psychology and behavior in the information age. The substantial impact of social media on the mental health of adolescents who engage in NSSI may exacerbate stress and negative outcomes even more than real-life events (Goreis et al., 2025).

Since 2021, the keywords “ecological momentary assessment” and “trajectory” have begun to burst, indicating that NSSI research is entering a more fine-grained phase of dynamic monitoring and behavioral prediction. The emergence of these terms not only reflects methodological updates but also suggests that researchers are attempting to integrate individual behavioral path modeling with early intervention, thereby advancing intervention strategies in precision and timeliness (Esposito et al., 2022; Hoelscher et al., 2025; Kudinova et al., 2024).

**Figure 6:**
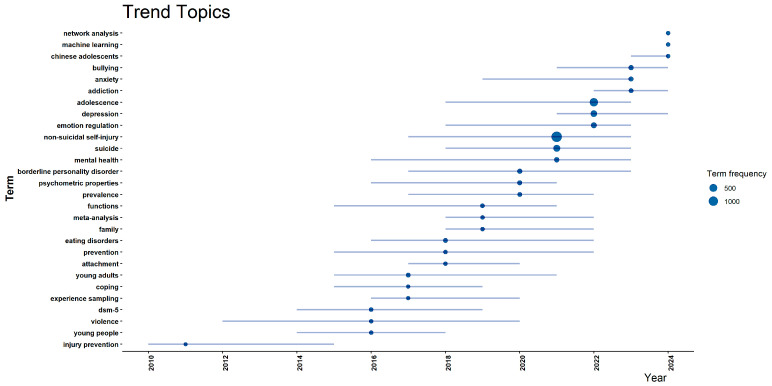
Temporal evolution map of adolescent NSSI research themes. Based on keyword evolution paths, it traces the migration of NSSI research themes over time, highlighting the shift in research focus from mood disorders to socio-ecological factors.

In conjunction with the thematic evolution map in Figure 5, the dynamic migration pathways among keywords are further revealed. The *x*-axis represents years, and the *y*-axis represents terms; circle size denotes term frequency, with larger circles indicating higher frequencies. This visualization helps identify the research foci and thematic evolution of NSSI across different periods. As shown in the Figure 6, early themes such as “injury prevention,” “young people,” and “violence” gradually evolved over time into themes related to psychological mechanisms and family functioning—“prevention,” “family,” “eating disorders,” and “coping”—suggesting that interventions for adolescent NSSI should attend to both family-level and individual-level factors (Kelada et al., 2018; Victor et al., 2019; Y. Wang et al., 2022). In recent years, sustained high frequencies of “non–suicidal self–injury,” “emotion regulation,” “depression,” and “adolescence” indicate the increasingly salient core role of mood disorders in the comorbidity mechanisms of NSSI. Related studies show that individuals exposed to emotional abuse in childhood are more likely to develop depressive symptoms and engage in NSSI (Lei et al., 2024). The occurrence of adolescent NSSI is closely linked to the use of emotion–regulation strategies and to family functioning (T. Zhao et al., 2021). Meanwhile, emerging themes such as “machine learning,” “network analysis,” and “Chinese adolescents” have surged since 2018. The use of machine learning models to identify adolescent mental health risk factors has garnered growing attention; their advantages in predicting comorbidity between depression and self-injury carry important practical value for developing prevention and intervention strategies targeting adolescent depression NSSI comorbidity (Huang et al., 2025).

This trend suggests the following:

(1) Artificial intelligence methods are being introduced into NSSI prediction and risk modeling research;
(2) Increasing attention is being paid to adolescents in China and other non–Western cultural contexts;
(3) Researchers are shifting from single-variable analyses toward psychological network construction and dynamic evolutionary models based on complex systems.

### 3.3. Co-Citation Analysis: Network Structure of Theoretical Foundations and Core Research Findings

#### 3.3.1. Highly Co-Cited Core References

**Figure 7:**
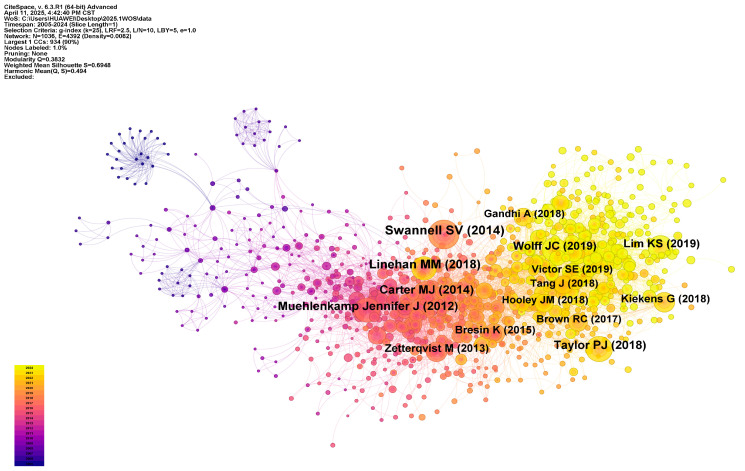
Co-cited references network.

Co-citation analysis is a classic bibliometric method for excavating a field’s knowledge core and intellectual lineage. In the NSSI domain, co-citation analysis reveals the knowledge structure and developmental trajectory of the field; as shown in Figure 7 and Table 2, the core co-cited works constitute important theoretical foundations.

In epidemiologic research on NSSI, Swannell and colleagues—through a systematic review of NSSI epidemiology—are the most frequently co-cited, underscoring the necessity of developing standardized methods in NSSI research and establishing NSSI as an independent object of study (Swannell et al., 2014). Lim and Plener likewise report high prevalence of non-suicidal self-injury and related self-harm in adolescents, with adolescence as the peak period. Lim highlights elevated prevalence in developing countries and in-school populations, revealing the impact of social and educational contexts on NSSI; from a longitudinal perspective, Plener shows that NSSI incidence rises during adolescence and declines into adulthood, with female sex and depressive symptoms as major predictors (Lim et al., 2019; Plener et al., 2015).

At the level of psychological mechanisms, emotion regulation provides a core lens for explaining adolescent NSSI. Linehan first proposed an emotion regulation-based intervention framework, which was foundational in clinical practice (Linehan, 1993). Building on this, Taylor et al. offer empirical support that most adolescents use NSSI to relieve negative affect, while interpersonal functions also motivate NSSI for some individuals, thereby extending the theory’s scope (Taylor et al., 2018). Hooley and Franklin’s “benefits–and–barriers” model further enriches understanding by positing that NSSI is attractive because of its affective benefits, though most people refrain due to inherent psychological barriers—an insight that provides new perspectives for risk assessment and intervention design (Hooley & Franklin, 2018). Wolff et al. further corroborate emotion dysregulation as a salient risk mechanism that is independent of age and sex, demonstrating cross-group applicability (Wolff et al., 2019).

Comorbidity between NSSI and other psychiatric disorders is likewise a central concern. Klonsky and colleagues point to the high comorbidity between NSSI and suicidal behavior; NSSI may reduce fear of suicide and increase capability, thereby functioning as a key antecedent to suicidal acts (Klonsky et al., 2013). A review by Cipriano et al. shows that NSSI is most prevalent among youth aged 12–14 and frequently co-occurs with borderline personality disorder and eating disorders (Cipriano et al., 2017). Wang et al. further identify a series of psychosocial risk factors strongly associated with NSSI—including psychiatric disorders, adverse childhood experiences, and bullying—emphasizing the interaction of psychopathology with environmental stress (Y. J. Wang et al., 2022). From an interpersonal perspective, Victor et al. (2019) and Tang et al. highlight that peer conflict, affective isolation, and social–cognitive difficulties—as well as sex, family educational level, and ethno-cultural background—can heighten adolescent NSSI risk, underscoring the roles of family and social relationships in comorbidity mechanisms (Tang et al., 2018; Victor et al., 2019).

Overall, research on adolescent NSSI is shifting from prevalence description toward multi-level exploration of psychological mechanisms, with growing attention to its intersecting comorbidities with emotion regulation difficulties, personality disorders, and suicide risk. This theoretical and empirical foundation provides solid support for future cross-cultural studies, mechanism validation, and the design of targeted interventions.

#### 3.3.2. Co-Citation Cluster Structure

**Figure 8:**
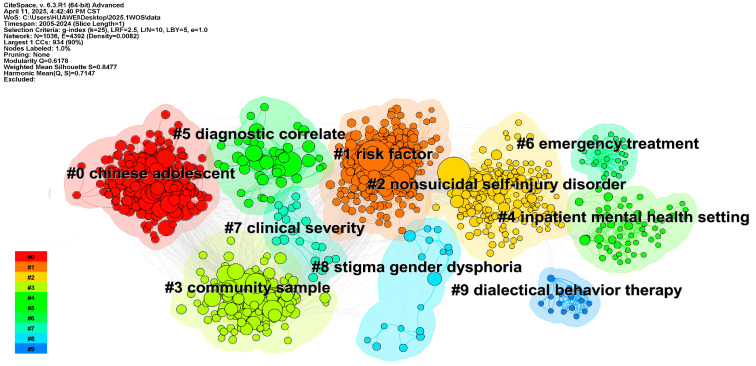
Reference co-citation network.

To reveal the knowledge structure and thematic distribution of adolescent non-suicidal self-injury (NSSI) research, we performed a cluster analysis of the literature co-citation network in CiteSpace6.3.R1. Ten high-quality clusters were identified (Modularity Q = 0.6178; Weighted Mean Silhouette = 0.8477), indicating good modularity and tight clustering (see Figure 8). The overall network comprises 1036 document nodes and 4392 co-citation links, with a density of 0.0082, reflecting strong inter-topic connectedness within the field. The ten clusters visualized cover multiple research directions—epidemiologic surveys, risk factor identification, diagnostic standard validation, mechanism model construction, and intervention applications—thereby illuminating the multidimensional evolutionary pathways of NSSI research and its close ties to psychiatric comorbidity. Analyses based on the co-citation map are as follows.

Cluster #0: “Chinese adolescent”. This cluster centers on NSSI among Chinese adolescents, emphasizing its relations to life events, anxiety, adverse childhood experiences, depression, sex differences, and mental health service access. Evidence shows extremely high NSSI prevalence among psychiatric inpatients in China; female sex, younger age, prior suicide attempts, more severe depressive symptoms, and greater exposure to negative life events are associated with higher risk. Life stress and negative affect play key roles in precipitating NSSI, and systematic screening and intervention strategies are especially important in psychiatric outpatient settings for NSSI (Xiao et al., 2023). This cluster also highlights attention to the characteristics of adolescent distress and self-injury within the Chinese cultural context, representing a notable trend toward the “Sinicization” of NSSI research. Prevention and intervention should consider culturally sensitive factors, such as gender discrimination, unrealistic achievement expectations, and inappropriate parenting, while also addressing emerging modern stressors—academic pressure, internet use, and romantic relationships. Integrating traditional and contemporary risk factors enables more precise assessment and intervention (X. Chen et al., 2021). Parental emotional warmth can reduce NSSI risk by decreasing involvement in bullying (Niu et al., 2024; Qin & Gan, 2024). Mental disorders, adverse childhood experiences, bullying, problem behaviors, female sex, low health literacy, and somatic symptoms all significantly increase NSSI risk, underscoring the critical roles of multidimensional psychological and social factors in NSSI onset (Y. J. Wang et al., 2022).

Cluster #1: “Risk factor”. This cluster examines risk factors for adolescent NSSI across biological, psychological, and environmental levels. Family dysfunction, trauma histories, and mental health problems (e.g., depression, anxiety) frequently emerge as key risks. Real-time monitoring technologies are proposed to elucidate the dynamic course of NSSI and its correlates (Kiekens et al., 2021). Regardless of age or sex, higher levels of emotion dysregulation are associated with greater NSSI risk across settings (Wolff et al., 2019). The “benefits–and–barriers” model further delineates NSSI risk factors and effective treatments (Hooley & Franklin, 2018). Understanding the ubiquity of NSSI functions is crucial for grasping its phenomenology and meeting diverse needs. Intrapersonal functions are most common and present in the majority of participants, supporting the central emotion regulation model and the value of interventions that enhance emotion regulation capacity. At the same time, interpersonal functions are endorsed by a substantial proportion of participants (Taylor et al., 2018).

Cluster #2: “Non–suicidal self–injury disorder.” Studies here focus on diagnostic criteria and clinical presentation of NSSI as a disorder, on distinguishing NSSI from suicidal behavior—some work positing NSSI as a key factor for suicide—and on clinical features and treatment. This literature underscores NSSI’s clinical significance, especially in relation to emotion regulation and impulse control. NSSI is a common, clinically meaningful behavior among adolescents and is typically associated with serious social, physical, and psychological consequences, with high comorbidity across conditions. Relations and distinctions between NSSI, suicidality, and borderline personality disorder are examined; introducing the concept of comorbidity, this body of work provides a comprehensive overview of adolescent NSSI epidemiology, comorbidities, clinical significance, functional mechanisms, and risk factors, with particular attention to distinctions and linkages involving internet use, suicidality, and BPD (In-Albon, 2015). DSM–5′s proposed NSSI disorder criteria have begun to be used to assess prevalence, yielding more precise estimates (Zetterqvist, 2015). Investigations of the extent to which NSSI contributes to later suicidal thoughts and behaviors (STBs), independent of shared risk factors, suggest that therapies enhancing a sense of meaning in life and fostering positive relationships—especially with parents—may be particularly effective in reducing suicide risk among adolescents with a history of NSSI (Klonsky et al., 2013; Whitlock et al., 2006).

Cluster #3: “Community sample.” This cluster focuses on studies conducted in community samples, typically outside clinical settings. The research addresses the prevalence of NSSI behaviors, psychological support and social influences within communities, and the effects of external factors—such as social media—on adolescent self-injury. There is growing concern about the prevalence of non-suicidal self-injury among adolescents; NSSI is highly prevalent among in-school youth and frequently co-occurs with suicidal ideation and suicide attempts. High-lethality NSSI, frequent alcohol use, and lifetime sexual experience have been identified as important screening indicators of potential suicide attempts. The findings underscore the need for early identification and intervention at the school level for the comorbidity of NSSI and suicidal behavior to reduce suicide risk among adolescents (Cheung et al., 2013).

Cluster #4: “Inpatient mental–health setting.” Prior research on social factors related to adolescent NSSI was limited by a narrow focus on specific interpersonal domains, cross-sectional designs, retrospective self-reports of childhood experiences, and failure to predict incident NSSI among adolescents not yet affected. In an urban sample of adolescent girls, both peer and parent factors predicted new-onset NSSI; however, in multivariable models that combined predictors, only peer factors were associated with subsequent NSSI. The results further indicated that both behavioral and cognitive/emotional indicators of interpersonal difficulties predicted NSSI onset. These findings highlight the relevance of family and peer relationships to the emergence of NSSI and have important implications for preventing onset among high-risk youth (Victor et al., 2019). Methodological factors affecting heterogeneity in prevalence estimates have been examined, alongside temporal trends and the estimation of overall international NSSI prevalence (Swannell et al., 2014). Regarding predictors, depressive symptoms and female sex are frequently reported as predictors of NSSI and DSH (Plener et al., 2015).

Cluster #5: “Diagnostic correlate.” This cluster documents diagnostic heterogeneity among adolescents who engage in NSSI and underscores the substantial overlap between NSSI and suicide attempts, examining diagnostic comorbidities and relationships with suicide attempts and bodily pain (Nock et al., 2006). It focuses on associations between adolescent NSSI and psychiatric diagnoses, particularly links with suicide attempts and with mood, personality, and substance use disorders. Although NSSI appeared in DSM–IV only as one diagnostic criterion for borderline personality disorder, a large body of research indicates that NSSI can exist independently of other mental disorders and serves a clear emotion regulation function (Klonsky, 2007; Whitlock et al., 2006). Features such as NSSI frequency, method diversity, and reduced pain sensitivity are important predictors of suicidal behavior. The view that emotion regulation is the primary function of NSSI has become a central starting point for research and intervention.

Cluster #6: “Emergency treatment.” This cluster captures the early developmental stage of research on emergency department (ED) interventions after self-injury and emphasizes the necessity of integrating mental health diagnostics into ED workflows. The ED is not only the first line of physical care but also a crucial window for identifying and intervening on mental disorders. This cluster has practical implications for strengthening adolescent crisis intervention policies, acute-phase suicide risk assessment tools, and multidisciplinary collaboration. Systematic mental health assessment in the ED following adolescent self-injury is essential and can improve detection of psychiatric disorders. Indeed, systematic mental health assessment after episodes of NSSI is critical; approximately half of young people are diagnosed with a mental disorder after a DSH event, and conducting structured assessment in the ED may increase detection rates (Olfson et al., 2005). Clinicians’ evaluations of NSSI are influenced by immediate risk factors such as current mental state and suicidal intent, whereas background risk factors (e.g., social adversity, psychiatric history) exert less influence. Training that emphasizes comprehensive appraisal of both immediate and background risk factors may improve the accuracy and timeliness of ED interventions (Cooper et al., 2003).

Cluster #7: “Clinical severity.” This cluster elucidates key mechanisms underlying clinical heterogeneity among adolescents with NSSI and emphasizes the need for tiered, stepped-care interventions tailored to individual clinical features. It underscores the importance of early identification of clinical severity markers (e.g., PPS, biomarkers, history of ACEs) for precision intervention and argues that NSSI alone should not serve as the sole metric of clinical recovery. Focusing on clinical samples, the cluster examines variables related to features of borderline personality disorder (BPD), emotion dysregulation, stress levels, and social dysfunction, highlighting NSSI’s clinical heterogeneity and its role as a risk marker for worsening psychopathology. For adolescents whose presentation is dominated by NSSI with milder BPD symptomatology, brief, low-intensity CDP (initial intervention) is typically sufficient; in contrast, those with more severe self/interpersonal dysfunction may require more comprehensive DBT–A. Three BPD diagnostic criteria can serve as threshold indicators within stepped care to determine whether escalation is warranted (Cavelti et al., 2024). Although most adolescents with NSSI show marked improvement within one year, full remission rates are low, and relapse/worsening risks remain high—particularly among those with lower baseline NSSI frequency. Inpatient treatment and depressive symptoms have been identified as key impediments to treatment response and remission, suggesting that outpatient care may be preferable in most cases. The pronounced heterogeneity of NSSI treatment outcomes underscores the importance of developing individualized strategies and of early identification of high-risk groups for relapse (Reichl et al., 2023). Among female adolescents, negative affect does not diminish after NSSI; instead, it intensifies over the short term and weakens attachment to mothers, underscoring the need for clinical psychoeducation addressing the transient nature of NSSI’s effects (Koenig et al., 2021). Dialectical Behavior Therapy for Adolescents (DBT–A) shows small-to-moderate effects in reducing self-harm and suicidal ideation, with pre–post-evaluations indicating significant improvements across all key outcomes (Kothgassner et al., 2021). A brief cognitive behavioral manual (CDP) achieves outcomes comparable to treatment as usual (TAU) in reducing adolescent NSSI and can expedite symptom relief; positioning CDP as the first line, low-intensity step in stepped care offers practical and cost-effective advantages (Kaess et al., 2020).

Cluster #8: “Stigma gender dysphoria.” This cluster focuses on gender dysphoria, sexual minority identities (e.g., LGBTQ+), and their relationships with NSSI. Identity conflict and social prejudice faced by sexual minority adolescents constitute key psychosocial risk factors for NSSI. Research emphasizes that conflicts in sexual orientation or gender identity, together with stigma experiences (e.g., discrimination, exclusion, shame), may indirectly elevate NSSI risk by impairing emotion regulation capacity. Sexual and gender minority populations are more likely to experience COVID-19-related depressive and anxious symptoms; however, they have received relatively limited research attention (Lorabi et al., 2023). Individuals identifying as sexual and gender minorities also report a higher prevalence of non-suicidal self-injury (NSSI). According to minority stress theory, the unique stressors associated with minoritized sexual orientation and/or gender identity increase the risk of adverse health outcomes. Accordingly, stress experienced by sexual and gender minorities may constitute an important risk factor for NSSI (Ahrenholtz et al., 2025). Sexual minority (LGB) adolescents face a significantly higher risk of NSSI than other groups, with the association primarily mediated by emotion regulation difficulties rather than concerns about sexuality per se—implicating interventions that strengthen emotion regulation among LGB youth (Fraser et al., 2018). Compared with heterosexual peers, LGB youth also show higher risks of NSSI and suicide-related behaviors (STBs), with risk strongest among those whose sexual attraction is more same-sex oriented; moreover, associations between NSSI (frequency, number of methods, number of functions) and STBs vary by type of sexual attraction (SA) (Tsypes et al., 2016). These findings underscore the importance of individualized, gender-sensitive risk assessment and intervention for sexual minority youth.

Cluster #9: “Dialectical behavior therapy.” This cluster centers on interventions for adolescent NSSI—especially the application and effectiveness of dialectical behavior therapy (DBT) in clinical practice. Regarding DBT implementation across community and inpatient settings, studies examine feasibility, cost-effectiveness, and efficacy in treating high-risk adolescents in different clinical contexts (e.g., community outpatient clinics, acute inpatient units). Multiple studies show that DBT significantly reduces self-injury, suicidal ideation, and related psychopathology such as depression, impulse–control problems, and BPD symptoms. Implementing DBT in community clinics is feasible and effective for high-risk adolescents, leading to notable reductions in suicide attempts, NSSI, suicidal ideation, and risk factors, including emotion dysregulation, depression, impulsivity, and BPD features (Berk et al., 2020). DBT outperforms supportive therapy in reducing suicide attempts, NSSI, and overall self-harm among adolescents, demonstrating clear clinical value and long-term potential for preventing suicidal behaviors in high-risk youth (McCauley et al., 2018). DBT–A appears to be an effective treatment with substantial potential for reducing adolescent self-harm and suicidal ideation by teaching core skills in emotion regulation, interpersonal effectiveness, and stress coping, thereby helping adolescents manage affective impulses and psychological pain and lowering the risk of extreme behaviors (Kothgassner et al., 2021).

Taken together, these highly co-cited clusters clarify interrelations between NSSI and psychiatric comorbidities and furnish new perspectives and directions for future research.

#### 3.3.3. Knowledge Evolution Pathways

**Figure 9:**
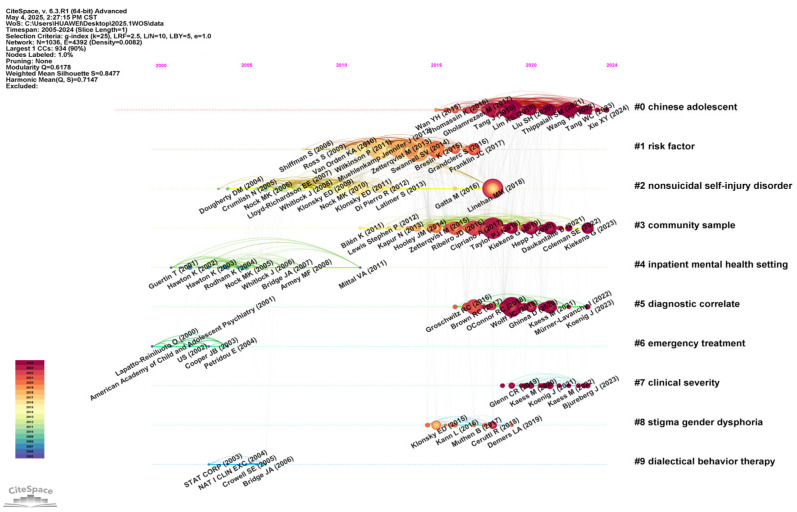
Co-citation timeline diagram.

The co-citation timeline further reveals the temporal characteristics of thematic evolution in this field (see Figure 9).

Phase I: Identification and Initial Construction (2000–2010).

At the outset, scholarship focused primarily on the clinical identification of non-suicidal self-injury (NSSI) and its relations to psychiatric disorders. Representative clusters in this stage are #5, “Diagnostic correlate”, and #6, “Emergency treatment,” which concentrated on comorbid features linking NSSI with conditions such as borderline personality disorder (BPD) and highlighted NSSI’s significance as a clinical symptom. Studies progressively clarified the central role of emotion regulation difficulties in the development of NSSI and identified the emergency department as a crucial setting for detecting NSSI and its underlying psychopathology. Although NSSI had not yet been defined as an independent disorder, research during this period established preliminary standards for recognizing its risk characteristics and functional purposes, laying a solid foundation for subsequent theoretical modeling and intervention work.

Phase II: Theoretical Expansion and Mechanism Modeling (2011–2015).

With the DSM–5′s proposed criteria for NSSI disorder, the research emphasis shifted from behavioral identification to mechanism building and functional analysis. Work in cluster #1, “Risk factor”, and cluster #2, “Non–suicidal self–injury disorder”, advanced the theoretical development of NSSI as a distinct diagnostic entity. Scholars formalized the “benefits–and–barriers” model to explain NSSI’s emotion regulation function and identified key risk factors—trauma exposure, depression, anxiety, and emotion dysregulation. Systematic examinations of comorbidity among NSSI and disorders such as depression, anxiety, and PTSD drove a transition from clinical observation to mechanism modeling. Researchers also stressed that, although NSSI and suicidal behavior are related, they remain relatively independent in motivation, frequency, and function. This stage marked a critical leap from “symptom identification” to “functional model construction,” providing a theoretical basis for precision intervention.

Phase III: Multidimensional Mechanism Deepening (2016–2020).

Building on attention to individual mechanisms, research extended to broader socio-ecological dimensions—family relationships, social support systems, and cultural differences. Cluster #3, “Community sample”, and cluster #4, “Inpatient mental health setting”, reflect this expanded lens, focusing on the prevalence of NSSI in non-clinical populations and on social determinants among hospitalized adolescents. The findings showed that, among in-school youth, NSSI commonly co-occurs with suicidal ideation, alcohol use, and interpersonal conflict, while factors such as family support and peer relations significantly predict behavioral occurrence. The addition of cross-cultural and gender difference studies underscored the ecological sensitivity of NSSI and its comorbidities. This phase signaled a move into integrated, multidimensional mechanism frameworks and supplied an ecological perspective for future intervention design.

Phase IV: Empirical Turn and Precision Intervention (2020–2024).

Recent work has simultaneously advanced empiricism, stratification, and cultural adaptation, shifting the focus from theory building to optimizing interventions and segmenting target populations. Research clusters include #0, “Chinese adolescent,” #7, “Clinical severity,” #9, “Dialectical behavior therapy,” and #8, “Stigma gender dysphoria,” collectively illustrating deepening along multiple dimensions. First, cultural sensitivity has become a key topic—particularly in the Chinese context—where gender role expectations, academic pressure, and family structure are highlighted as influences on NSSI. Second, clinical pathways have grown more refined. Stepped care strategies align with individual severity (e.g., DBT–A for high-risk adolescents; streamlined cognitive behavioral therapy for medium-to-low-risk groups). Third, sexual minority youth have drawn increasing attention as a high-risk subgroup, with social stigma and emotion regulation difficulties identified as core mechanisms. This empirical turn has strengthened the identification and treatment of high-risk comorbid groups and laid the groundwork for individualized, multidimensional intervention systems.

Phase V: Future Trends and Research Frontiers.

Adolescent NSSI research is expected to further advance toward cross-disciplinary integration, intelligent detection, and fine-grained elucidation of comorbidity mechanisms. Continued progress in neurobiology, ecological behavior-tracking technologies, and AI algorithms will facilitate big data-based dynamic identification systems capable of real-time monitoring and early warning for high-risk individuals and behavioral trajectories. Network analysis approaches can integrate NSSI with common disorders (anxiety, depression, PTSD) to uncover latent interaction mechanisms and key mediators, thereby enhancing the specificity and systems-level targeting of interventions. Cross-cultural comparisons will deepen, informing comorbidity patterns and intervention strategies across diverse social contexts. Research on sexual minority and socially marginalized youth will broaden the social dimension of comorbidity structures, emphasizing the cumulative effects of structural stress and identity conflict. The combined influences of culture, gender identity, and clinical features underscore the complexity of NSSI comorbidity networks and the need for more precise treatment matching. Integrating neuroimaging, biomarkers, and AI-based monitoring will support predictive, adjustable intervention models and propel a shift from static diagnosis to dynamic prevention and care. Ultimately, adolescent NSSI research will achieve deeper integration from mechanism understanding to clinical practice along the pathway of “comorbidity networks—ecological mechanisms—individualized interventions.”

## 4. Discussion

### 4.1. Current Challenges

Building on the above analyses and framework, the field currently faces the following challenges that warrant scholarly attention.

(1) Unclear boundary between the independence and comorbidity of NSSI. Although the DSM–5 has proposed “non–suicidal self–injury disorder” as a diagnostic entity, NSSI in clinical practice frequently co-occurs with depressive disorders, anxiety disorders, and borderline personality disorder (BPD), and a unified set of identification criteria and a structural framework is still lacking. Causal relations, interaction pathways, and potential mediators within comorbidity mechanisms remain insufficiently modeled. Psychiatric diagnoses are contested constructs that may be vague, overlapping, and susceptible to circularity. We note ongoing debates on the adverse consequences of diagnostic labels (Veldmeijer et al., 2025). Because most existing studies rely on cross-sectional data and self-report measures, it is difficult to conduct dynamic analyses of comorbidity network structure or to generate recommendations for targeted intervention (Y. Chen et al., 2023; Gillies et al., 2018).

(2) A multi-layered evolution from theory → mechanism → intervention, but limited attention to network complexity under multimorbidity. While research hotspots show a progressive trajectory, dominant topics still cluster around traditional psychological variables, such as emotion regulation, family functioning, and trauma exposure, with relatively few systematic examinations of the complexity of network structures under co-occurring disorders. Although intervention approaches (e.g., DBT–A) continue to be refined, their adaptability to comorbid presentations has not been fully tested, and real-world effectiveness is significantly moderated by cultural and gender factors (Kaess et al., 2020; Kothgassner et al., 2021).

(3) Cultural context limitations and “cultural mismatch.” Mainstream theories are largely derived from Euro–American contexts and have tended to overlook how family structure, educational pressure, and gender roles in China and other non-Western societies shape the emergence and course of NSSI. Prevention, assessment, and intervention should more fully incorporate culturally sensitive factors. For example, gender stereotypes and discrimination, unrealistic achievement expectations, and controlling parenting practices may impose latent psychological stress on adolescents. In addition, emerging stressors in contemporary society—intensifying academic competition, immersive internet use, and affective volatility in romantic relationships—should be incorporated into intervention planning to address the diverse antecedents of adolescent NSSI (X. Chen et al., 2021; Xiao et al., 2023). The resulting “cultural mismatch” weakens the effectiveness and accessibility of current intervention models; against the backdrop of increasingly salient conflicts between adolescents’ plural identity formation and social expectations, this research gap is particularly conspicuous.

### 4.2. Future Research Characteristics

Research on adolescent non-suicidal self-injury (NSSI) continues to grow, driving the ongoing evolution of hotspots and the emergence of new themes. Accordingly, researchers urgently need a systematic and accurate framework for understanding future directions and theoretical characteristics of NSSI and its psychiatric comorbidity networks. Theoretically, drawing on the knowledge map constructed in this study and the challenges identified herein, we infer the following research characteristics.

(1) A comorbidity network perspective will become mainstream. Analyses centered on single comorbidity variables are giving way to more systemic, structurally oriented research. Future work will more frequently employ symptom network analysis, multilevel regression modeling, and dynamic tracking to elucidate mediating mechanisms linking NSSI with anxiety, depression, adverse childhood experiences, post-traumatic stress disorder (PTSD), and eating disorders (Lei et al., 2024; Niu et al., 2024; H. Zhao & Zhou, 2024). Clarifying path-specific patterns of risk accumulation will, in turn, provide a theoretical basis for individualized interventions.

(2) Cross-cultural research will emerge as a new hotspot. Cross-cultural comparisons will inform the construction of culturally adaptive intervention models. Because the social meanings, functional orientations, and behavioral patterns of NSSI differ between Eastern and Western cultures (Westers, 2024), future studies should reconstruct conceptual models of NSSI comorbidity and intervention pathways from a cultural psychology perspective (X. Chen et al., 2021), enhancing the theoretical autonomy of local research and advancing a cultural turn in theory building.

(3) Methodology will evolve toward a multimodal dynamic paradigm. Leveraging multimodal data collection—neuroimaging (e.g., fMRI), wearable devices, and ecological momentary assessment (EMA)—in combination with machine learning and predictive modeling, researchers can build dynamic monitoring systems across time and contexts, enabling a shift from “behavior identification” to “risk prediction” (Gan et al., 2025; Herzog et al., 2022; Liao et al., 2024; Nicolaou et al., 2025; Sun et al., 2024). In addition, longitudinal tracking and natural experiment designs will facilitate causal inference models, strengthening the scientific identification of comorbidity mechanisms.

### 4.3. Comparison Discussions

A comparative discussion facilitates a deeper understanding of the field; this constitutes the novelty and contribution of our work. By comparing this study with prior reviews related to non-suicidal self-injury (NSSI), we identify the following differences.

(1) Core focus on the “NSSI–psychiatric comorbidity network.” We move beyond the limitations of treating NSSI as merely an accessory symptom of borderline personality disorder (BPD) or as an isolated behavioral phenomenon and instead emphasize its independence, complexity, and interactivity within the adolescent psychopathology spectrum. Unlike earlier reviews that typically centered on a single variable (e.g., self-injury motives, emotion regulation, family influences) or a specific intervention pathway, this study integrates, from a macro-structural perspective, the relational architecture and interactions between NSSI and multiple psychiatric comorbidities, thereby offering readers a more systematic theoretical lens.

(2) Visualization-driven mapping of knowledge structures and evolutionary trends. Using co-citation clustering, keyword co-occurrence, and timeline mapping, we delineate the field’s knowledge structure and its evolution, furnishing scholars with a clear, intuitive research landscape and hotspot trajectory. In contrast, existing reviews largely rely on traditional manual theme aggregation, which introduces subjectivity and structural limitations. Through data-driven clustering, we not only identify the focal directions of different research groups but also capture the shift from emotion regulation theory toward symptom network modeling.

(3) Problem-driven response and forward-looking agenda. Beyond addressing current core challenges—such as unclear mechanisms within the NSSI comorbidity structure, methodological monocultures, constrained cultural applicability, and insufficient attention to marginalized groups—we propose future-facing research characteristics: comorbidity network modeling, deepening of cross-cultural perspectives, the introduction of multimodal methodologies, and the expansion of structural, society-level intervention pathways. By comparison, although existing reviews provide strong empirical support for risk factors and intervention effectiveness, they remain limited in structural visualization, hotspot evolution detection, and cross-topic integration, and they lack a knowledge graph-based structural framework to support a holistic understanding of the NSSI comorbidity domain and the construction of a forward-looking research agenda.

## 5. Conclusions

Using CiteSpace6.3.R1, VOSviewer 1.6.20, and R 4.3.3, we analyzed annual publication trends, keyword co-occurrence, keyword clustering, burst-term detection, and co-citation patterns to systematically portray this field’s developmental trajectory, research hotspots, and frontier trends, and we preliminarily explored its structural comorbidity network and prospective evolution pathways. The main findings are as follows.

(1) Annual publication trends. Since 2014, research on adolescent non-suicidal self-injury (NSSI) has shown sustained growth. Year-over-year increases in publication counts reflect the field’s rising visibility and importance within the scholarly community.

(2) Keyword analytics. High-frequency keywords, “non–suicidal self–injury,” “adolescence,” “depression,” and “suicide”, constitute the core terminological system of the field; terms such as “borderline personality disorder” and “child maltreatment” also exhibit high betweenness centrality, serving as critical bridges across subfields. Keyword clustering further indicates that hotspots concentrate on comorbidity mechanisms of mood disorders; features related to personality disorders; impacts of childhood trauma; cross-presentations of psychopathological behaviors; and the development of measurement tools and dynamic assessment techniques. Timezone evolution mapping and burst-term detection show a thematic progression from early explorations of NSSI per se and its relation to suicide, to mid-stage emphases on emotion regulation, social support, and environmental stressors, and, in recent years, to growing attention on social–media effects, psychological mechanisms in sexual minority youth, dynamic measurement tools, and machine learning models. While mood and personality disorders have matured into core themes with relatively solid theoretical foundations, digital technologies, dynamic tracking methods, and culture-informed NSSI research are emerging directions with strong growth potential.

(3) Co-citation and clustering results. Core highly cited works center on NSSI epidemiology, emotion regulation mechanisms, and identification of comorbid disorders, establishing a robust theoretical and empirical base. Cluster structure analyses further reveal thematic distribution and hotspot evolution. Current research largely spans five directions: (i) risk factor identification and mechanism modeling, emphasizing interactions among emotion regulation, psychological trauma, and social stress; (ii) diagnostic standards and clinical heterogeneity, advancing recognition of NSSI as a distinct disorder and refining intervention strategies; (iii) community samples and cultural sensitivity, addressing expression patterns and intervention needs across cultures and populations; (iv) optimization of clinical treatment pathways, particularly stepped care strategies and the efficacy of dialectical behavior therapy (DBT); and (v) psychological risks and stigma effects among sexual minority groups, highlighting the roles of structural stress and identity conflict in NSSI comorbidity mechanisms. In terms of knowledge evolution pathways, NSSI research can be divided into five stages: an initial phase focused on clinical features and psychiatric comorbidity; a middle phase centered on functional model construction and mechanism exploration; a subsequent expansion into socio-ecological and cultural adaptation; a move toward stratified and individualized intervention strategies; and, currently, an empirical turn supported by big data, artificial intelligence, and ecological behavior tracking that stresses dynamic identification, precision prevention, and cross-cultural comparison. Looking ahead, the field will emphasize cross-disciplinary integration, construction of psychiatric comorbidity networks, and differentiated interventions for high-risk groups. With integrated applications of neuroimaging, biomarkers, and intelligent technologies, NSSI research is poised to shift from static diagnosis to dynamic prediction and to achieve deep integration of clinical efficacy and public health intervention along the pathway of “comorbidity identification—mechanism elucidation—multidimensional intervention.”

The innovations of this study lie mainly in three aspects as follows.

(1) Knowledge level. From multiple dimensions—keyword co-occurrence, clustering analysis, burst-term detection, and literature co-citation networks—we systematically traced the evolutionary pathway of research on adolescent non-suicidal self-injury (NSSI) and psychiatric comorbidity, constructing a multidimensional knowledge map of the field.

(2) Theoretical level. By identifying the evolutionary structure of research themes and the core works embedded in the clustering network, we established the theoretical fulcrums and logical developmental framework of adolescent NSSI research, deepening understanding of emotion regulation models, risk factor mechanisms, and patterns of multicomorbidity.

(3) Practical level. We revealed a staged shift in NSSI scholarship—from clinical identification to mechanism modeling and then to precision intervention—and focused on developments in culturally sensitive populations and intervention pathways, thereby providing theoretical grounding and methodological references for future intervention design and cross-cultural inquiry.

Nonetheless, several limitations should be noted.

First, self-citation bias is unavoidable. Some authors’ citation counts may be elevated by self-citation, which could, to some extent, affect the objectivity and representativeness of the co-citation analysis. Second, the findings have temporal limitations. Because our data extend only through December 2024, newly emerging results were not included and should be incorporated in future updates to refine the conclusions. Third, single-source data was used. We relied solely on the Web of Science Core Collection, which may omit relevant studies indexed in Scopus, PubMed, or CNKI, introducing potential regional bias and database constraints. Finally, language scope must be considered. The analysis included only English-language publications. Although English is the primary language of international scholarship, excluding studies in Chinese, Spanish, and other languages may result in incomplete coverage of NSSI research in particular regions.

Future research can be extended in several ways: remove self-citations and conduct comparative analyses to enhance reliability; incorporate multiple databases to broaden sample coverage; and include and compare the non-English literature to improve cross-cultural applicability. We actively invite scholars in this field to contribute objective, up-to-date insights, thereby minimizing the potential adverse impact of researcher subjectivity on the analysis. Although longitudinal studies remain relatively scarce, they are on an upward trajectory. Moving forward, we will closely monitor and continually track new work in this area and dynamically update the knowledge map to enhance the timeliness and representativeness of the findings.

## Figures and Tables

**Table 1:** High-frequency keywords.

**Ranking**	**Frequency**	**Centrality**	**Year**	**Keyword**
1	949	0.03	2005	non-suicidal self-injury
2	725	0.02	2005	adolescence
3	650	0.01	2006	suicide
4	592	0.02	2005	prevalence
5	589	0.02	2007	harm
6	471	0.01	2005	risk factors
7	359	0.02	2007	depression
8	341	0.01	2006	behavior
9	270	0.05	2010	emotion regulation
10	252	0	2009	meta-analysis
11	164	0.06	2010	mental health
12	163	0.04	2010	child maltreatment
13	163	0.05	2007	community sample
14	146	0.04	2011	behaviors
15	141	0.06	2006	borderline personality disorder
16	126	0.03	2013	validity
17	125	0.04	2010	reliability
18	116	0.02	2015	symptoms
19	112	0.05	2014	stress
20	109	0.02	2014	psychometric properties

**Table 2:** Top 20 co-cited references.

**Ranking**	**Count**	**Year**	**Reference**
1	135	2014	(Swannell et al., 2014)
2	123	2018	(Linehan, 1993)
3	103	2018	(Taylor et al., 2018)
4	100	2012	(Muehlenkamp et al., 2012)
5	98	2014	(Carter, 2014)
6	89	2019	(Lim et al., 2019)
7	80	2019	(Wolff et al., 2019)
8	72	2018	(Kiekens et al., 2018)
9	64	2015	(Bresin & Schoenleber, 2015)
10	63	2013	(Zetterqvist et al., 2013)
11	58	2019	(Victor et al., 2019)
12	57	2018	(Hooley & Franklin, 2018)
13	56	2018	(Gandhi et al., 2018)
14	54	2017	(Brown & Plener, 2017)
15	53	2018	(Tang et al., 2018)
16	52	2013	(Klonsky et al., 2013)
17	52	2015	(Plener et al., 2015)
18	51	2017	(Cipriano et al., 2017)
19	51	2012	(Hamza et al., 2012)
20	50	2022	(Y. J. Wang et al., 2022)

## Data Availability

No new data were created or analyzed in this study.
